# Comparative study of modified vs. traditional sacroiliac screw techniques in day type II crescent fracture dislocation of the pelvis

**DOI:** 10.3389/fsurg.2025.1674184

**Published:** 2025-12-11

**Authors:** Renjie Li, Xiaopan Wang, Leyu Liu, Jianzhong Guan, Peishuai Zhao, Xiaotian Chen, Min Wu

**Affiliations:** Clinical Laboratory, The First Affiliated Hospital of Bengbu Medical College, Bengbu, China

**Keywords:** pelvis, fractures, closed, fracture fixation, internal, posterior screw fixation, sacroiliac

## Abstract

**Background:**

The use of traditional sacroiliac screws in the treatment of Day type II crescent fracture-dislocation of the pelvis (CFDP) is often associated with insufficient screw anchorage and compression due to the short iliac segment of the screw and the proximity of the entry point to the fracture line. This study aims to evaluate a modified sacroiliac screw technique designed to address these limitations.

**Methods:**

In this retrospective comparative study, we analyzed 44 patients with Day type II CFDP who underwent surgical treatment between January 2019 and June 2023. Based on the sacroiliac screw technique applied, patients were divided into a modified group (*n* = 21) and a control group (*n* = 23). We compared the sacroiliac screw-related metrics, the quality of reduction assessed by Matta score, and clinical outcomes evaluated using the Majeed functional score and the visual analogue scale (VAS) for pain.

**Results:**

All patients successfully underwent surgery. The modified group exhibited a significantly longer iliac segment of the sacroiliac screw compared to the control group (3.71 ± 0.85 cm vs. 2.12 ± 0.47 cm, *P* < 0.001). The distance from the screw entry point to the iliac fracture line was greater in the modified group (3.31 ± 0.88 cm vs. 1.22 ± 0.64 cm, *P* < 0.001). Reduction quality assessed by Matta score one week postoperatively was superior in the modified group: excellent in 12, good in 7, fair in 2, and poor in 0 cases; vs. excellent in 6, good in 12, fair in 5, and poor in 0 cases in the control group (*P* < 0.05). At the final follow-up, the modified group showed better VAS scores for sacroiliac joint pain than the control group (*P* < 0.05). No significant difference was observed in Majeed functional scores between the two groups (*P* = 0.568).

**Conclusion:**

The modified sacroiliac screw technique significantly extends the iliac segment length, enhances cortical bone engagement, reduces the risk of entry point proximity to the fracture line, and improves screw stability and reduction quality. However, these findings require validation through larger prospective and biomechanical studies to further assess long-term efficacy and applicability.

## 1 Introduction

Crescent fracture-dislocation of the pelvis (CFDP) is a subtype of lateral compression (LC) pelvic injury, accounting for approximately 12% of lateral compression pelvic injuries (1). It refers to a fracture line caused by lateral compression that passes through the sacroiliac joint and extends superiorly and laterally to the iliac wing, forming a characteristic crescent-shaped iliac fragment that remains firmly attached to the sacrum via the sacroiliac joint, accompanied by sacroiliac joint dislocation (2, 3). Day (1) classified CFDP into three types based on the extent of sacroiliac joint involvement: Type I involves less than one-third of the sacroiliac joint, resulting in a large posterior crescentic iliac fragment; Type II involves the middle third of the sacroiliac joint, resulting in a medium-sized posterior crescentic iliac fragment; and Type III involves more than two-thirds of the sacroiliac joint, resulting in a small posterior crescentic iliac fragment.

Day type II CFDP accounts for about half of all CFDP cases. Surgical intervention is currently recommended to restore pelvic ring stability and facilitate early rehabilitation. Traditional surgical methods primarily involve open reduction and internal fixation (ORIF) via anterior or posterior approaches (4–6). However, these approaches are associated with significant trauma, substantial blood loss, and a high rate of complications, which can adversely affect patient outcomes. With the widespread adoption of minimally invasive concepts and the application of navigation devices such as robots, percutaneous screw fixation techniques have gained popularity (7, 8). Percutaneous sacroiliac screw fixation combined with posterior iliac screws for Day type II pelvic crescent fractures not only provides good biomechanical stability but also reduces the complications associated with open reduction and internal fixation, making it increasingly favored and applied by clinicians (9).

However, in clinical practice, we have identified certain limitations associated with the use of traditional sacroiliac screw fixation for treating Day type II CFDP: On one hand, the screw entry point may be in close proximity to or even directly located on the iliac fracture line, which compromises the compressive reduction effect of the screw and increases the risk of reduction loss. On the other hand, the relatively short iliac segment of the screw trajectory reduces screw purchase, potentially leading to insufficient fixation strength, particularly in patients with osteoporosis.

**Figure 1:**
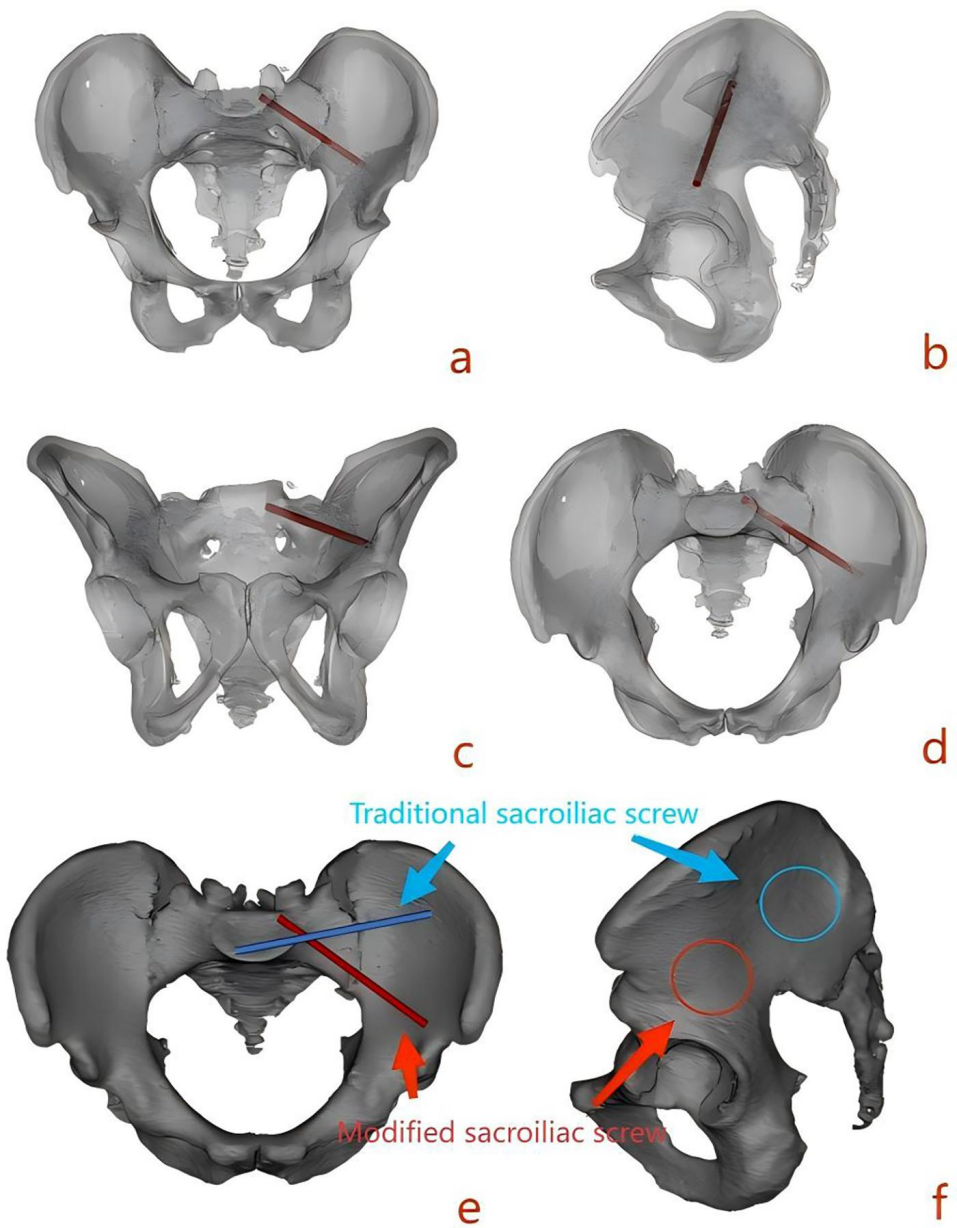
**(a)** Modified sacroiliac screw front view. **(b)** Modified sacroiliac screw lateral view. **(c)** Modified sacroiliac screw outlet view. **(d)** Modified sacroiliac screw inlet view. **(e)** Comparison of the modified and traditional sacroiliac screws on a pelvic inlet view. **(f)** Difference in the entry point region between the modified and traditional sacroiliac screws on a pelvic lateral view.

In Day type II CFDP surgery, the iliac entry point of the sacroiliac screw must avoid the fracture line while achieving a sufficiently long bony channel to ensure biomechanical strength. Inspired by the “S2 alar-iliac screw” (S2AIS) technique (10–12), we designed a new sacroiliac screw trajectory: by adjusting the entry point and direction, the screw achieves a longer intra-iliac channel while staying away from the iliac fracture line. However, whether this modified approach is truly superior to traditional screw placement lacks evidence-based support (Figure 1).

Therefore, this study retrospectively compared cases of Day type II CFDP treated with modified vs. traditional sacroiliac screw fixation, systematically evaluating the operability, screw parameters, and early clinical outcomes of both techniques to clarify the safety advantages and application value of the modified technique.

## 2 Patients and methods

This study is a retrospective cohort study. The study protocol adhered to the principles of the Declaration of Helsinki and was approved by the Ethics Committee of Bengbu Medical College (No. BYYFY-2018YJS180). All patients and their families provided informed consent regarding the use of the surgical techniques described in this study before surgery.

A total of 44 patients with Day type II CFDP who met the criteria were ultimately enrolled from January 2019 and June 2023. All surgeries were performed by the same chief physician with over 10 years of experience in pelvic fracture treatment to control for the impact of surgeon variability on the results.

The inclusion criteria were: (i) radiographically confirmed Day type II pelvic crescent fracture-dislocation; (ii) complete preoperative and postoperative imaging data; (iii) age ≥18 years.

The exclusion criteria were: (i) congenital developmental or acquired diseases causing abnormal bony structure of the pelvic ring; (ii) concomitant acetabular fractures requiring surgical management; (iii) clinical follow-up time <6 months; (iv) pathological pelvic fractures (13, 14).

**Figure 2:**
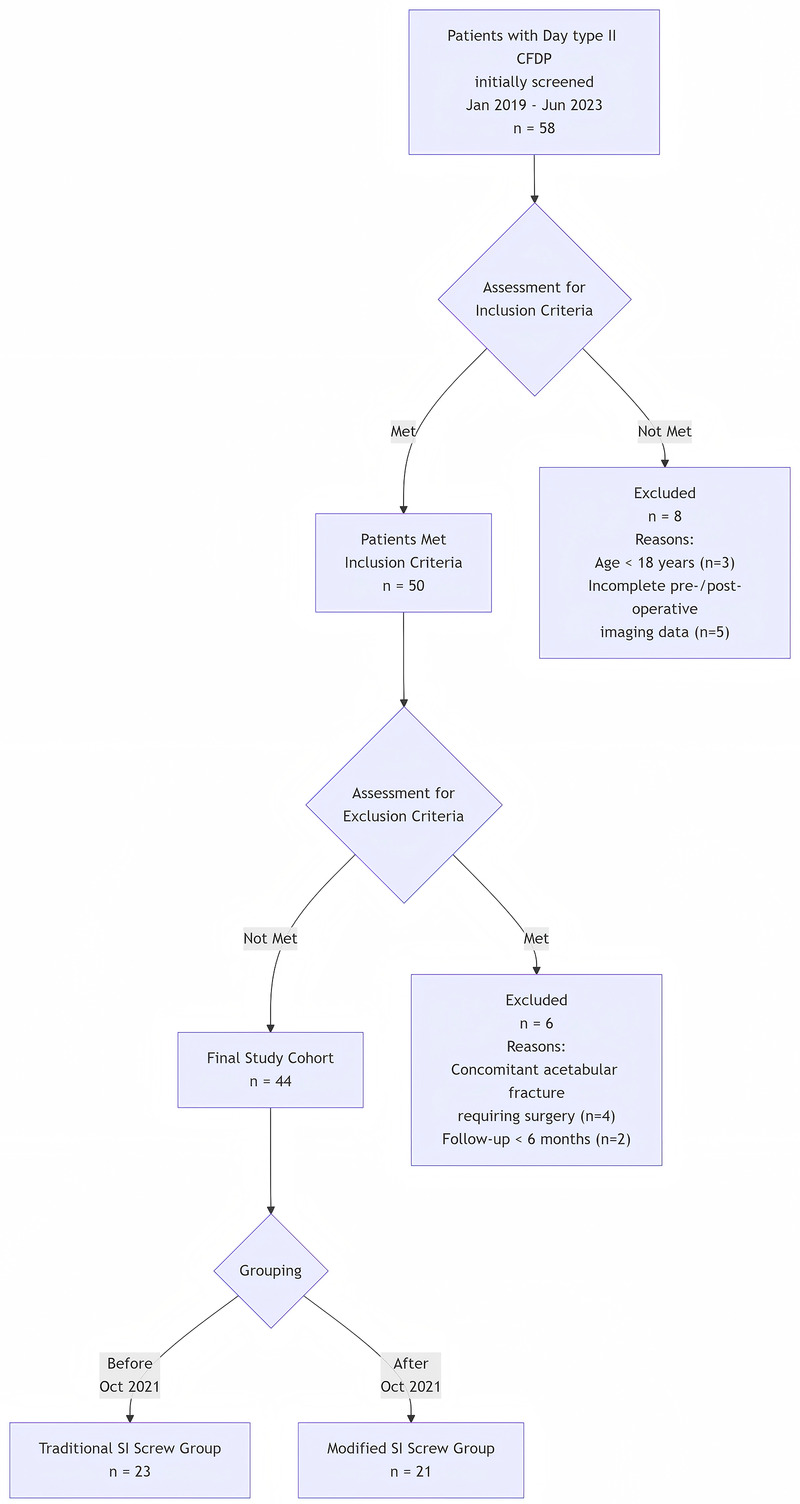
Patient enrollment flowchart.

Finally, 44 patients who met the criteria were included in this study. The choice of internal fixation was based on the surgical technique preference during different periods: Before October 2021: The team had not mastered the standardized procedure for the modified sacroiliac screw placement technique; during this period, traditional sacroiliac screw internal fixation was used. After October 2021: The team underwent specialized training and mastered the modified sacroiliac screw placement technique; during this period, the modified sacroiliac screw internal fixation was used (Figure 2).

Based on the sacroiliac screw technique actually used during surgery, the 44 patients were divided into two groups: the “modified group” (*n* = 21) using the modified sacroiliac screw technique and the “control group” (*n* = 23) using the traditional sacroiliac screw technique.

The intraoperative navigation assistance system used was the TiRobot system.

### 2.1 Surgical treatment

#### 2.1.1 Modified group

After satisfactory anesthesia, the patient was placed in the supine position on a radiolucent carbon fiber operating table. We first fixed the iliac fracture with an LC-II screw, then proceeded with the placement of the modified sacroiliac screw.

**Figure 3:**
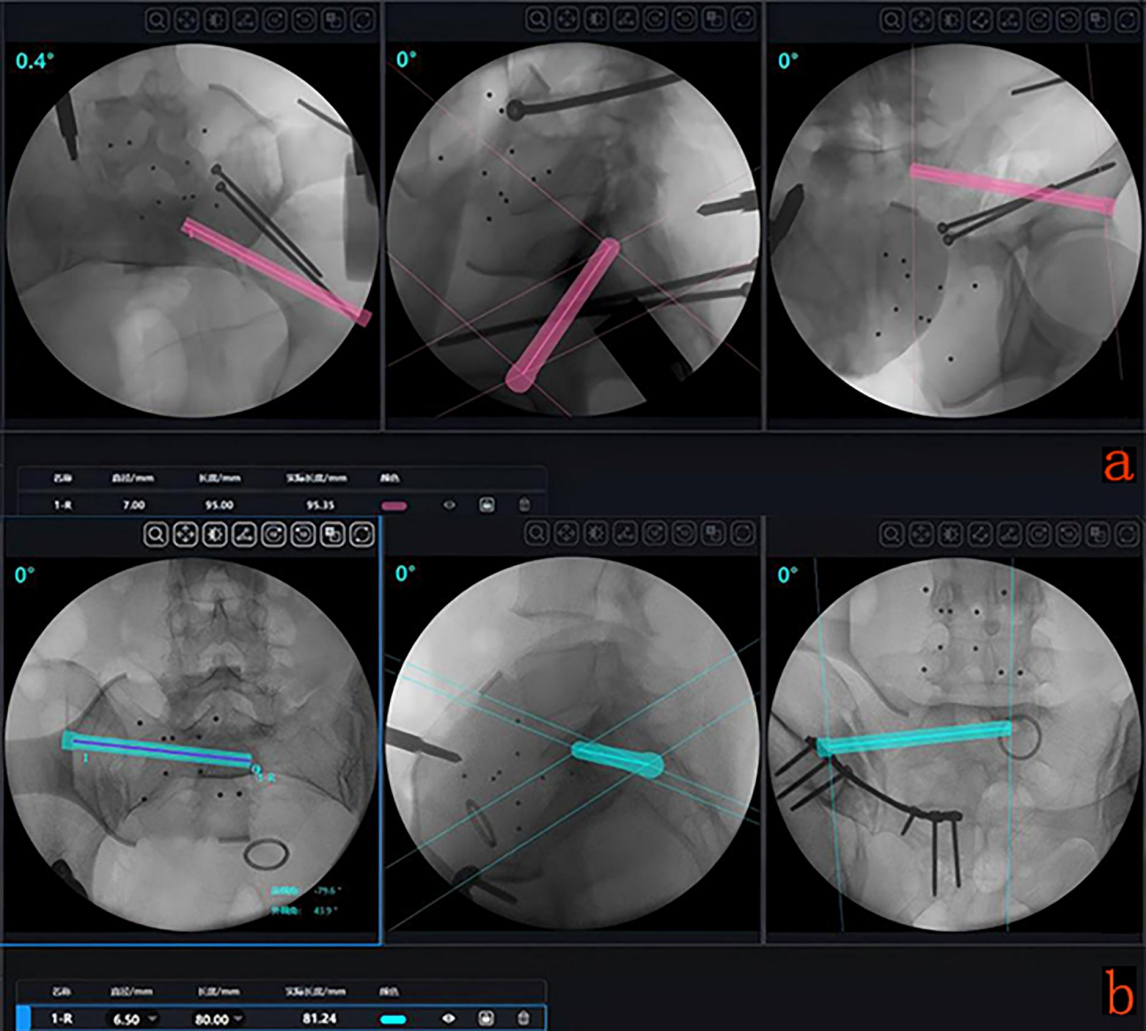
**(a)** Intraoperative planning images for the modified sacroiliac screw. **(b)** Intraoperative planning images for the traditional sacroiliac screw.

We attached the tracker to the contralateral anterior superior iliac spine, used a C-arm to obtain pelvic inlet, outlet, and lateral view images, and uploaded the images to the main control computer. We planned the screw trajectory on the inlet and outlet views and verified screw safety on the pelvic lateral view. During planning, on the inlet view, the screw entry point was located on the anterolateral aspect of the ilium, and the exit point was medial to the superior articular process of S1. On the outlet view, the entry point was located at the superior rim of the acetabulum, directed towards the S1 vertebral body, passing between the S1 anterior sacral foramen and the sacral alar slope. On the lateral view, when the screw entered the sacroiliac joint, the superior border should not exceed the iliac cortical density line and the sacral alar slope, and the inferior border should not exceed the greater sciatic notch. After planning was completed, the robotic arm was manipulated to the designated position, a guide wire was inserted, the length was measured, and a cannulated screw was inserted (Figure 3).

Finally, depending on the type of anterior pelvic ring fracture, INFIX, plating, or anterior column screws were used to treat the anterior pelvic ring injury (15).

#### 2.1.2 Control group

The fracture reduction method was the same as in the modified group.

When planning the traditional sacroiliac screw, on the inlet view, the screw entry point was located on the posterolateral aspect of the ilium, and the exit point was near the anterior edge of the S1 vertebral body. On the outlet view, the entry point was located at the superior rim of the acetabulum, directed towards the S1 vertebral body, passing between the S1 anterior sacral foramen and the sacral alar slope. Screw safety was verified on the pelvic lateral view (16). After planning was completed, the robotic arm was manipulated to the designated position, a guide wire was inserted, the length was measured, and a cannulated screw was inserted (Figure 3).

Finally, an appropriate fixation method was used to treat the anterior pelvic ring.

### 2.2 Postoperative management

All patients received routine anticoagulants postoperatively to prevent deep vein thrombosis (DVT). Within one week after surgery, multi-angle x-rays (including front, inlet, outlet, lateral, and iliac wing views) and thin-slice CT (0.625 mm) scans were performed to assess fracture reduction and screw position. Regular x-rays were taken at 6 weeks, 3 months, 6 months, 12 months, and 24 months postoperatively to evaluate patient recovery and guide functional exercises. At 8 weeks postoperatively, patients were allowed to ambulate with crutches. At 12 weeks, depending on fracture healing, patients were allowed to attempt walking without crutches.

All patients were followed up for a minimum of 6 months (mean follow-up duration: 14.5 ± 4.2 months). Follow-up was complete for all enrolled patients, with no loss to follow-up.

### 2.3 Outcome measurements

We pre-specified and collected the following variables: patient demographics (gender, age), injury characteristics (cause of injury, fracture classification according to the Tile system), perioperative metrics (operative time, intraoperative blood loss, intraoperative radiation exposure time), and follow-up duration.

The primary endpoint was the quality of fracture reduction, assessed during the first postoperative week using the Matta criteria on pelvic radiographs (anteroposterior, inlet, and outlet views). The maximal displacement was measured and graded as follows: excellent (<4 mm), good (4–10 mm), fair (11–20 mm), or poor (>20 mm) (17).

**Figure 4:**
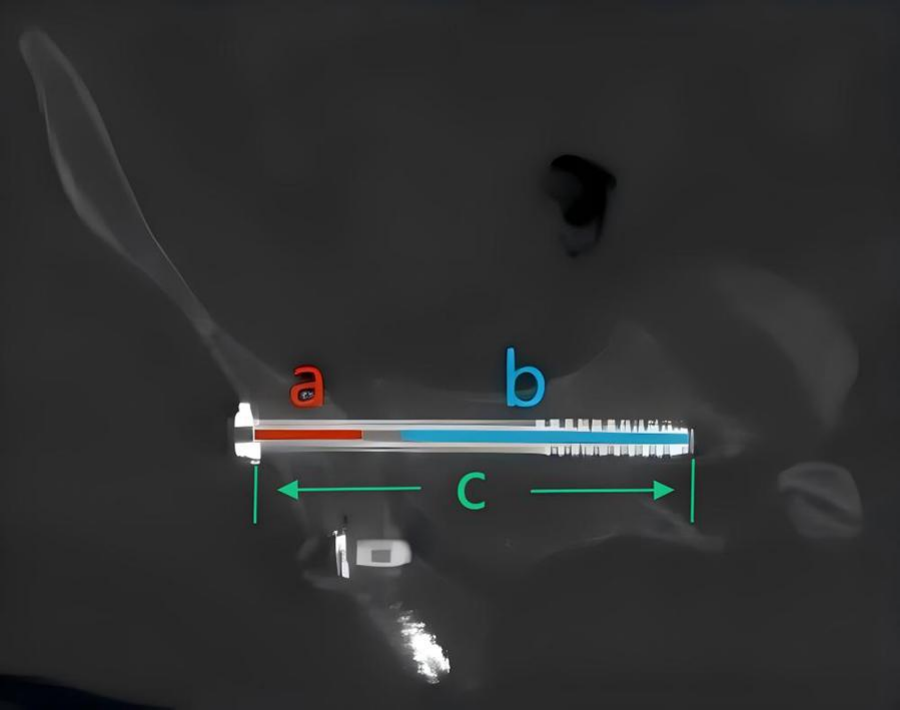
Measurement of sacroiliac screw-related metrics: **(a)** length of iliac segment for sacroiliac screws. **(b)** Length of sacral segment for sacroiliac screws. **(c)** Total length of sacroiliac screws.

Key secondary endpoints included: 1. Implant-related metrics: measured on postoperative computed tomography (CT), including (i) the total length of the sacroiliac screw, (ii) the length of its iliac segment, (iii) the length of its sacral segment, and (iv) the shortest distance from the screw's entry point to the iliac fracture line (The shortest distance from the center of the screw tail to the iliac fracture line was measured on three-dimensional reconstructed views using the medical imaging software Mimics Medical 21.0). 2. Functional outcome: evaluated at the final follow-up using the Majeed score (18), which incorporates pain, work capacity, sitting and standing capability, sexual function, assisted ambulation, gait, and walking distance. Scores were categorized as excellent (≥85), good (70–84), fair (55–69), or poor (<55). 3. Pain assessment: The intensity of sacroiliac joint–specific pain was quantified at the final follow-up using the visual analogue scale (VAS). 4. Safety profile: All postoperative complications (e.g., surgical site infection, neurovascular injury, deep vein thrombosis, loss of reduction) were documented (Figure 4).

### 2.4 Statistical analysis

All statistical analyses were performed using SPSS version 22.0 (IBM Corp., USA). Measurement data (age, operative time, intraoperative blood loss, fluoroscopy time, total screw length, iliac segment length, sacral segment length, distance from entry point to fracture line) were tested for normality using the Shapiro–Wilk test. Data conforming to a normal distribution are expressed as mean ± standard deviation (*x¯* ± *s*), and intergroup comparisons were made using independent samples *t*-tests. Count data (gender, cause of injury, fracture classification, Matta reduction quality grade, Majeed functional score grade, VAS pain score grade, complications) are expressed as frequency (*n*, %), and intergroup comparisons were made using the *χ*^2^ test or Fisher's exact test. For intergroup comparisons, continuous variables are reported as mean difference (MD) with 95% confidence interval (CI), and categorical variables are reported as odds ratio (OR) or relative risk (RR) with 95% CI. A *p*-value <0.05 was considered statistically significant.

## 3 Results

A total of 44 patients were included in the final analysis, with 21 in the modified group and 23 in the control group. There were no statistically significant differences between the two groups in terms of gender, age, cause of injury, fracture classification, as well as follow-up duration (*P* > 0.05), indicating comparability.

There were no statistically significant differences between the two groups in operative time, intraoperative blood loss, or intraoperative radiation exposure time (*P* > 0.05) (Table 1).

**Table 1:** Patient demographics, injury characteristics, and perioperative metrics.

Variable	Modified group (*n* = 21)	Control group (*n* = 23)	*P*
Age, years (mean ± SD)	48.6 ± 10.2	51.3 ± 11.5	0.412
Gender, *n* (%)			0.765
Male	14 (66.7%)	14 (60.9%)	
Female	7 (33.3%)	9 (39.1%)	
Cause of injury, *n* (%)			0.841
Traffic accident	15 (71.4%)	17 (73.9%)	
Fall from height	6 (28.6%)	6 (26.1%)	
Tile classification, *n* (%)			0.896
B	17 (81.0%)	20 (87.0%)	
C	4 (19.0%)	3 (13.0%)	
Operative time, min (mean ± SD)	95.2 ± 21.8	102.5 ± 25.4	0.321
Blood loss, mL (mean ± SD)	180.5 ± 50.3	195.8 ± 61.7	0.378
Radiation time, s (mean ± SD)	28.4 ± 9.1	31.6 ± 10.5	0.285

### 3.1 Reduction quality (primary endpoint)

According to the postoperative Matta score criteria (17), reduction quality in the modified group was excellent in 12 cases, good in 7 cases, fair in 2 cases, with no poor results. In the control group, reduction quality was excellent in 6 cases, good in 12 cases, fair in 5 cases, with no poor results. The reduction quality in the modified group was superior to that in the control group, and the difference was statistically significant (*P* < 0.05).

### 3.2 Comparison of sacroiliac screw-related metrics

All screws were successfully inserted. The average total length of the sacroiliac screws in the modified group was longer than that in the control group, but the difference was not statistically significant (*P* > 0.05). However, the average length of the iliac segment of the screw in the modified group was significantly longer than that in the control group [(3.71 ± 0.85) cm vs. (2.15 ± 0.60) cm; *P* < 0.001]. The average shortest distance from the screw entry point to the iliac fracture line in the modified group was also significantly greater than that in the control group [(3.31 ± 0.88) cm vs. (0.91 ± 0.55) cm; *P* < 0.001]. There was no statistically significant difference in the average length of the sacral segment of the screw between the two groups (*P* > 0.05) (Table 2).

**Table 2:** Comparison of sacroiliac screw-related metrics.

Parameter	Modified group (*n* = 21)	Control group (*n* = 23)	MD (95% CI)	*P*
Total screw length, cm	9.44 ± 0.76	8.47 ± 0.61	0.97 (0.55 to 1.39)	0.261
Iliac segment length, cm	3.71 ± 0.85	2.12 ± 0.47	1.59 (1.14 to 2.04)	<0.001
Sacral segment length, cm	5.65 ± 0.79	6.09 ± 0.70	−0.44 (−0.90 to 0.02)	0.057
Distance to fracture line, cm	3.31 ± 0.88	1.22 ± 0.64	2.09 (1.64 to 2.54)	<0.001

### 3.3 Comparison of clinical and functional outcomes

#### 3.3.1 Majeed score

At the final follow-up, the Majeed functional score (18) in the modified group was excellent in 11 cases, good in 6 cases, and fair in 4 cases, with an excellent-good rate of 81.0%. The rate in the control group was 73.9%. The difference in functional scores between the two groups was not statistically significant (*P* = 0.568).

#### 3.3.2 VAS score

At the final follow-up, the VAS score for sacroiliac joint pain in the modified group was 0 in 10 cases, <3 in 7 cases, and 4–6 in 4 cases. In the control group, it was 0 in 8 cases, <3 in 9 cases, and 4–6 in 6 cases. Among the 44 patients, 34 had a VAS score below 3 at the final follow-up, with an overall pain relief rate of 77.3%.

### 3.4 Complications

**Figure 5:**
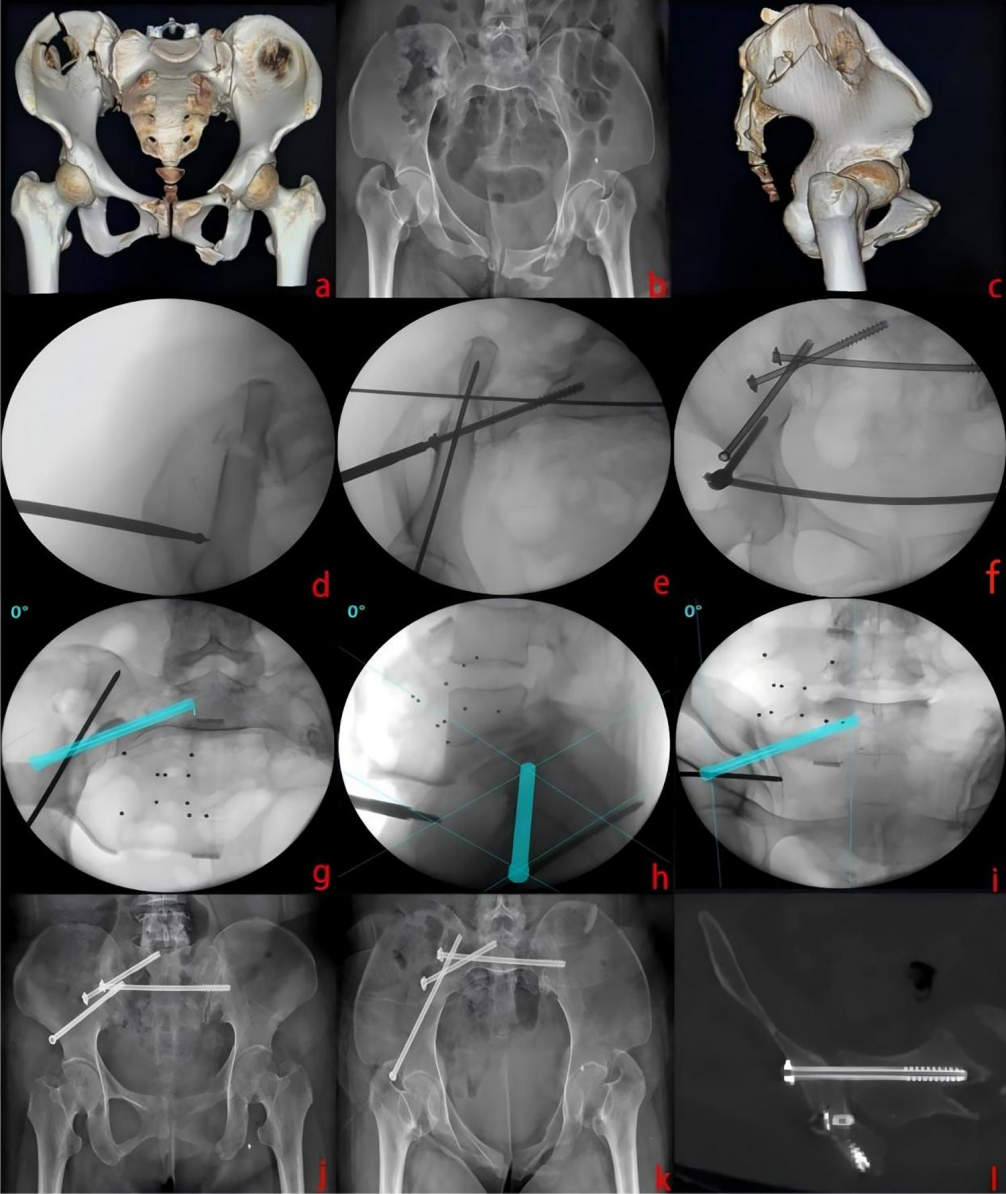
Pre-operative CT and x-ray images of a 44-year-old female patient with a Day type II CFDP **(a–c)**. Through a 1-cm incision, fracture reduction was achieved with an apex cone on the iliac outer table, followed by fixation with a guide pin and subsequent placement of modified SI, LC-II, and S2 screws, with anterior stability provided by INFIX **(d–f)**. Intraoperative modified sacroiliac screw planning images **(g–i)**. One-year follow-up imaging confirmed anatomical reduction, good fracture healing, and intact implants **(j–l)**.

**Figure 6:**
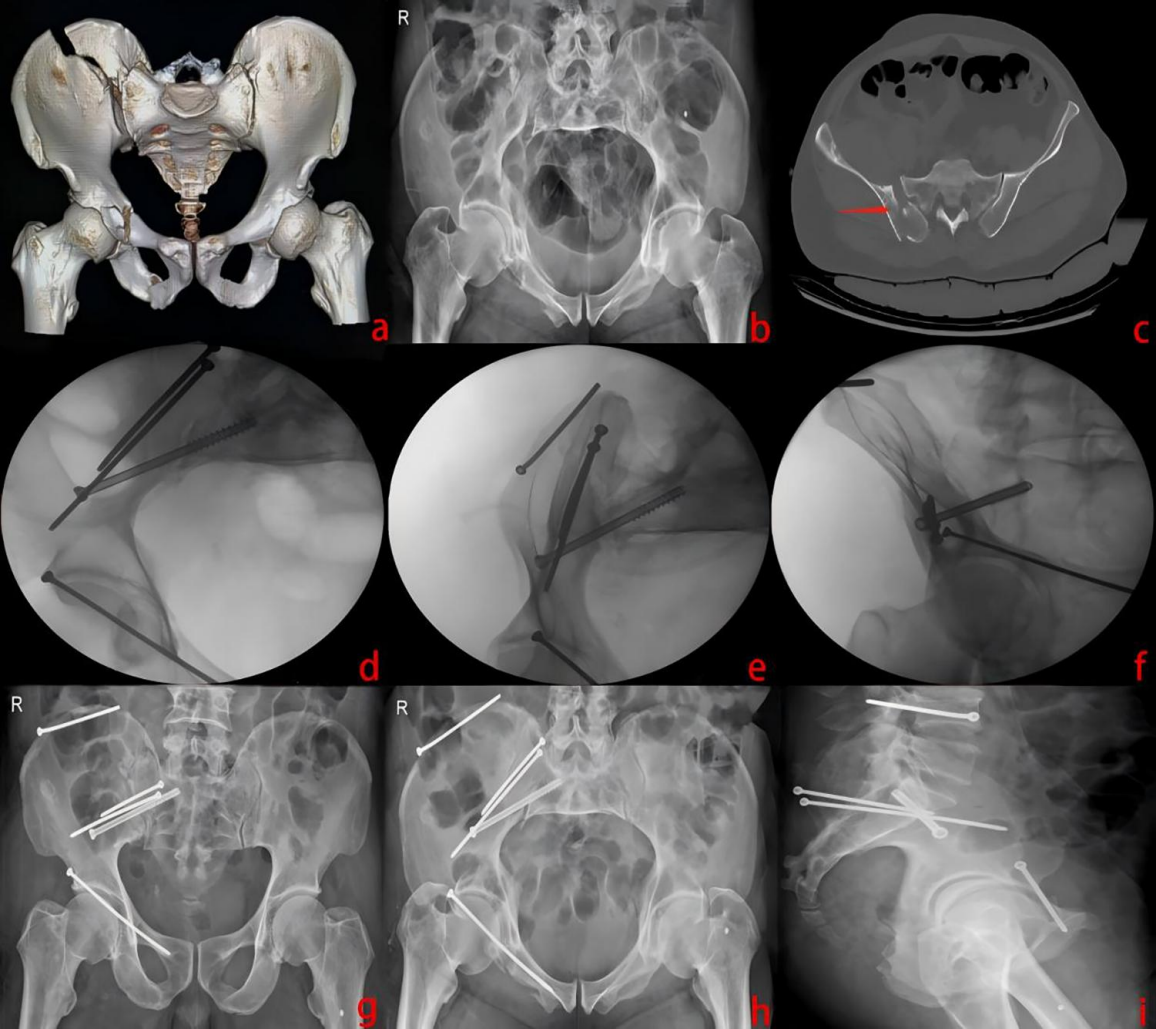
Pre-operative CT and x-ray images of a 58-year-old male patient with a Day type II CFDP **(a–c)**. Surgery was performed with the patient in the prone position. Small incisions were made over the posterior superior iliac spine (PSIS) and the right iliac crest to reduce the iliac fracture. Two LC-II screws were inserted through the PSIS for fixation, followed by placement of a modified sacroiliac screw. Finally, the right superior pubic ramus was fixed with an anterior column screw **(d–f)**. Postoperative pelvic radiographs demonstrated satisfactory fracture reduction and appropriate implant positions **(g–i)**.

In the modified group, 3 patients developed postoperative DVT, resulting in a complication rate of 14.29% (3/21). In the control group, there were 2 cases of DVT, 1 case of surgical site infection, and 1 case where the sacroiliac screw entered the sacral foramen (without associated neurological symptoms). Additionally, we found that the sacroiliac screw entry point was located on the iliac fracture line in 3 patients. One of these patients experienced loss of sacroiliac joint reduction during follow-up, but fortunately, it did not significantly affect the patient's life and work, so no further intervention was performed. The overall complication rate in this group was 17.39% (4/23) (Figures 5, 6) (Table 3).

**Table 3:** Comparison of clinical and functional outcomes.

Outcome measure	Modified group (*n* = 21)	Control group (*n* = 23)	*P*
Reduction quality, *n* (%)			0.035
Excellent (<4 mm)	12 (57.1%)	6 (26.1%)	
Good (4–10 mm)	7 (33.3%)	12 (52.2%)	
Fair (11–20 mm)	2 (9.5%)	5 (21.7%)	
Poor (>20 mm)	0 (0%)	0 (0%)	
Majeed score, *n* (%)			0.568
Excellent (≥85)	11 (52.4%)	11 (47.8%)	
Good (70–84)	6 (28.6%)	8 (34.8%)	
Fair (55–69)	4 (19.0%)	4 (17.4%)	
Poor (<55)	0 (0%)	0 (0%)	
VAS score, *n* (%)			0.036
0 (No pain)	10 (47.6%)	8 (34.8%)	
1–3 (Mild)	7 (33.3%)	9 (39.1%)	
4–6 (Moderate)	4 (19.0%)	6 (26.1%)	
Complications, *n* (%)	3 (14.3%)	4 (17.4%)	1.000
Deep vein thrombosis	3 (14.3%)	2 (8.7%)	
Surgical site infection	0 (0%)	1 (4.3%)	
Loss of reduction	0 (0%)	1 (4.3%)	

## 4 Discussion

This retrospective analysis compared the clinical and imaging outcomes of the modified sacroiliac screw technique with the traditional sacroiliac screw technique in the treatment of Day type II CFDP. The results showed that the modified group was superior to the control group in terms of the length of the iliac segment of the screw, the distance from the screw entry point to the fracture line, and the postoperative fracture reduction quality, indicating that this technique has advantages in enhancing screw holding power and improving reduction quality. From an anatomical and biomechanical perspective, the modified sacroiliac screw, by optimizing the entry point location and screw trajectory, not only effectively avoids the issue of reduced holding power caused by the entry point being on the fracture line but also significantly improves screw stability in osteoporotic bone through a longer intra-iliac screw channel and engagement of both medial and lateral cortices, thereby creating more favorable mechanical conditions for early postoperative functional exercise (13).

Burgess et al. (19) and Manson et al. (20) classified pelvic ring injuries into three main categories based on the mechanism of injury: anterior-posterior compression (APC), lateral compression (LC), and vertical shear (VS). Day type II crescent fracture-dislocation is an LC type II injury. Previous studies mostly suggested that the ligaments in the lower part of the sacrum remain intact, resulting only in rotational instability but vertical stability (3, 21). However, Burgess et al. (19) pointed out that when the pelvis is subjected to vertical violence, injuries similar to crescent fractures can occur, and significant vertical displacement of the anterior part of the ilium can be seen on pelvic front view. Zong et al. (4) used vertical displacement of the sacroiliac joint exceeding 1 cm as the criterion for judging pelvic vertical stability. In their study of 31 patients with crescent fractures, they found 27 cases with pelvic rotational instability, 4 of which also had vertical instability. In our study, 7 out of the 44 patients with Day type II crescent fractures had significant vertical displacement of the sacroiliac joint, and all these 7 patients resulted from traffic accidents. We believe this is because traffic accident violence is multi-directional and high-energy, so Day type II crescent fracture patients can also have pelvic vertical displacement. For these patients, we performed preoperative supracondylar femoral traction, which helped reduce the sacroiliac joint subluxation to some extent. Additionally, during surgery, vertical traction reduction should be coordinated while correcting rotational displacement (22).

Compared to the traditional sacroiliac screw, the entry point of the modified sacroiliac screw is located on the anterolateral aspect of the superior acetabular rim, and the exit point is medial to the superior articular process of S1. This channel design draws on the long-channel concept of the S2AIS screw (10–12), but the entry point is more anterolateral, avoiding the problem of the traditional posterolateral entry point easily overlapping with the fracture line. Ma et al. (23) proposed, through anatomical studies on a large number of 1:1 normal 3D-printed pelvises, that a normal adult pelvis has an anterior sacroiliac screw channel. The medial wall of this channel is formed by the pelvic arcuate line, the lateral wall by the posterolateral sacral ala and the lateral part of the ilium, the superior wall by the sacral promontory and the floor of the greater pelvis, and the inferior wall by the line between the sacral foramen and the greater sciatic notch. Postoperative CT scans of all patients in the modified group showed that the screws were located within the bony channel and did not enter the acetabulum or sacral canal, which to some extent reflects the safety of the modified sacroiliac screw channel (24).

The design of the modified sacroiliac screw demonstrates significant biomechanical advantages. By changing the screw entry point and insertion direction, the technique achieves an extended screw path within the ilium. The data from this study show that the average length of the iliac segment of the sacroiliac screw in the modified group was significantly longer than that in the control group [(3.71 ± 0.85) cm vs. (2.15 ± 0.60) cm], and the entry point was farther from the fracture line [(3.31 ± 0.88) cm vs. (0.91 ± 0.55) cm]. This design not only effectively avoids issues such as ineffective compression caused by the entry point being on the fracture line but also increases fixation strength through greater cortical purchase. Especially for patients with osteoporosis (25), the longer bony channel and bicortical purchase enhance pull-out strength and rotational stability, providing a reliable mechanical foundation for early postoperative functional exercise (12).

Regarding reduction quality, the Matta score was better in the modified group. We attribute this advantage primarily to two aspects: first, the entry point of the modified sacroiliac screw effectively avoids the fracture line, ensuring the screw can fully exert its compression effect; second, the point of direct compression of the screw is closer to the area of most significant anterior dislocation of the sacroiliac joint, achieving more direct and effective compression reduction of the joint surface (13). This biomechanical advantage is difficult to achieve with the traditional posterolateral entry approach.

Although there was no significant difference in the Majeed functional scores between the two groups at the final follow-up, the modified group had lower VAS pain scores, suggesting potential benefits of this technique in improving local symptoms and patient comfort. It is worth noting that the lack of difference in functional scores may be related to the limited sample size, insufficient follow-up time, or the sensitivity of the assessment tool to stability improvements.

Although the overall complication rate was not statistically different between groups (*P* = 1.000), the nature of the complications differed markedly. Complications in the control group, such as screw malposition into the sacral foramen and entry point placement on the fracture line (which led to one case of loss of reduction), are directly technical and inherent to the limitations of the traditional screw trajectory. In contrast, complications in the modified group (e.g., deep vein thrombosis) were systemic and unrelated to the implant technique itself.

In addition, the use of intraoperative navigation equipment provided important guarantees for accurate screw placement, not only improving surgical safety but also reducing the number of intraoperative fluoroscopies and radiation exposure (7, 8).

It is important to note that this study has several limitations. First, the sample size is small, and it is a single-center retrospective study, which may introduce selection bias and technical bias (all surgeries were performed by the same surgeon). Second, there is a lack of biomechanical experimental validation; the holding power of the modified sacroiliac screw needs further clarification through *in vitro* experiments. Third, the sacral segment channel of the modified sacroiliac screw is slightly shorter. When pelvic vertical displacement is significant, an S2 transsacral screw might be needed to enhance fixation. Furthermore, although the mean follow-up time was not significantly different between the groups, comparing functional scores at fixed time points (e.g., 6 months, 1 year postoperatively) might further reduce the potential influence of the time factor. This is an aspect that could be improved in future prospective studies. Finally, this technique has a steep learning curve. Although the use of robots can help us place screws more accurately, this may be difficult to widely implement in primary hospitals (26).

## 5 Conclusion

In summary, the modified sacroiliac screw technique demonstrates promising application value for treating Day type II CFDP. By optimizing the entry point and trajectory, it effectively avoids the fracture line and enhances fixation stability through a longer iliac segment and improved cortical purchase. This technique proves particularly beneficial for osteoporotic patients and represents a valuable alternative or complement to traditional methods. However, future prospective multicenter studies are required to further validate its long-term efficacy and broader applicability.

## Data Availability

The raw data supporting the conclusions of this article will be made available by the authors, without undue reservation.
